# A pilot intervention trial to reduce the use of post-procedural antimicrobials after common endourologic surgeries

**DOI:** 10.1017/ice.2024.172

**Published:** 2025-01

**Authors:** Daniel J. Livorsi, Vignesh T. Packiam, Qianyi Shi, Steven Y. Alberding, Knute D. Carter, James A. Brown, James B. Mason, Jeffrey P. Weiss, Ryan L. Steinberg

**Affiliations:** 1 Iowa City Veterans’ Administration Health Care System, Iowa City, IA, USA; 2 Department of Medicine, University of Iowa Carver College of Medicine, Iowa City, IA, USA; 3 Rutgers Cancer Institute of New Jersey, New Brunswick, NJ, USA; 4 Department of Biostatistics, University of Iowa, Iowa City, IA, USA; 5 Department of Urology, University of Iowa Carver College of Medicine, Iowa City, IA, USA; 6 Malcolm Randall Department of Veterans Affairs Medical Center, Gainesville, FL, USA; 7 Department of Urology, University of Florida College of Medicine, Gainesville, FL, USA; 8 Brooklyn Veterans Affairs Medical Center, Brooklyn, NY, USA; 9 Department of Urology, SUNY Downstate Health Science University, Brooklyn, NY, USA

## Abstract

**Objective:**

Post-procedural antimicrobial prophylaxis is not recommended by professional guidelines but is commonly prescribed. We sought to reduce use of post-procedural antimicrobials after common endoscopic urologic procedures.

**Design:**

A before-after, quasi-experimental trial with a baseline (July 2020–June 2022), an implementation (July 2022), and an intervention period (August 2022–July 2023).

**Setting:**

Three participating medical centers.

**Intervention:**

We assessed the effect of a bundled intervention on excess post-procedural antimicrobial use (*ie*, antimicrobial use on post-procedural day 1) after three types of endoscopic urologic procedures: ureteroscopy and transurethral resection of bladder tumor or prostate. The intervention consisted of education, local champion(s), and audit-and-feedback of data on the frequency of post-procedural antimicrobial-prescribing.

**Results:**

1,272 procedures were performed across all 3 sites at baseline compared to 525 during the intervention period; 644 (50.6%) patients received excess post-procedural antimicrobials during the baseline period compared to 216 (41.1%) during the intervention period. There was no change in the use of post-procedural antimicrobials at sites 1 and 2 between the baseline and intervention periods. At site 3, the odds of prescribing a post-procedural antimicrobial significantly decreased during the intervention period relative to the baseline time trend (0.09; 95% CI 0.02–0.45). There was no significant increase in post-procedural unplanned visits at any of the sites.

**Conclusions:**

Implementation of a bundled intervention was associated with reduced post-procedural antimicrobial use at one of three sites, with no increase in complications. These findings demonstrate both the safety and challenge of guideline implementation for optimal perioperative antimicrobial prophylaxis.

This trial was registered on clinicaltrials.gov, NCT04196777.

## Introduction

Reducing the use of unnecessary antimicrobials is a key strategy for slowing the emergence of antimicrobial resistance. Opportunities to reduce unnecessary antimicrobial-prescribing exist across all of healthcare, including in surgical patients.

Extended courses of antimicrobial prophylaxis in the post-operative period are common in surgical patients.^1,2^ Within the field of urology, prior studies have estimated that a third of patients receive antimicrobial prophylaxis after a urologic procedure even though this practice is no longer recommended by the American Urological Association’s (AUA) guidelines.^3–5^ The vast majority of these post-procedural antimicrobials were deemed unnecessary by manual chart adjudication in one retrospective study across 5 hospitals.^3^ The frequent use of post-procedural antimicrobials remains a concern because this practice places patients at increased risk of antimicrobial-related harm, particularly *Clostridioides difficile* infection (CDI).^4,6–10^

To reduce post-procedural antimicrobial use, audit-and-feedback of performance data has proven effective.^11,12^ However, these prior initiatives have largely excluded urologic procedures. The purpose of this multicenter study was to evaluate whether a bundled intervention, which included audit-and-feedback, could safely reduce the use of post-procedural antimicrobials in patients undergoing common endoscopic urologic procedures.

## Methods

### Study design

We performed a pre-post, quasi-experimental study across three Veterans Affairs medical centers (VAMCs) to evaluate whether a bundled intervention can decrease the frequency of post-procedural antimicrobial-prescribing. We defined a baseline period (July 2020–June 2022), an implementation period (July 2022), and an intervention period (August 2022–July 2023). This trial was registered on clinicaltrials.gov, NCT04196777.

### Site selection

Eligible VAMCs needed to perform the three procedures of interest: ureteroscopy, transurethral resection of the prostate (TURP), and transurethral resection of a bladder tumor (TURBT). We chose these procedures because they are common endoscopic procedures without a skin incision. In addition, eligible sites needed to average ≥ 75 qualifying procedures per year and have a frequency of post-procedural antimicrobial use that was in the highest tertile for all sites.^3,4^

### Recruitment

We sent an e-mail invitation to the Chief of Urology at 13 of 35 eligible sites identified based on the above criteria. Four sites expressed interest in participation. The main study team (D.J.L., R.L.S., and V.T.P.) subsequently held a video-conference call with each of these sites to explain the project details and confirm interest. Three sites ultimately agreed to enroll, *ie,* to adopt the intervention.

### Site characteristics

Site 1 included two geographically-distinct procedural locations. Urology residents were involved in procedures at site 1’s main hospital (266 acute-care beds) while surgeries at the second location for site 1 were only performed by staff physicians. Sites 2 and 3 were smaller (< 100 acute-care beds) and had urology residents involved in all procedures.

### Intervention

Based on our review of the literature, we anticipated that there would be several barriers to prescribing post-procedural antimicrobials less frequently. These barriers included skepticism about guidelines, fear of adverse consequences, competing priorities for the urologists’ time, and lack of knowledge about the potential adverse effects of antimicrobial use.^13–15^ Many of these barriers, including skepticism, were observed during our initial meeting with the Chief or Urology at each site prior to site enrollment in the study. Suspected facilitators of using less post-procedural antimicrobials included a desire among urologists to improve outcomes in their patients and a desire to practice in line with community norms.^15^

To address these barriers and to leverage these facilitators, the intervention we designed used the implementation strategies of education, local champions and audit-and-feedback with peer-to-peer comparisons. Below is a time line of how these strategies were implemented:.

- In July 2022, the main study team held a virtual meeting with each site to review the AUA’s guidelines on antimicrobial prophylaxis,^5^ potential harms of extending antimicrobial prophylaxis beyond a single dose,^4,8^ and baseline (prior 2 yr) data on the frequency of post-procedural antimicrobial use for the three above-mentioned endourologic procedures at that site with comparisons to all VAMCs performing at least 75 of the qualifying procedures per year. Attendance at this meeting varied across sites: 15 of 15 staff urologists and nurse practitioners at site 1; 2 of 5 staff urologists at site 2; and 15 of 26 staff and resident urologists at site 3. Urology residents did not attend the introductory call at either sites 1 or 2.
- For the next 12 months, the Chief of Urology at each participating site was sent an e-mail every 2 months from D.J.L. with updated data on their site’s use of post-procedural antimicrobials with comparisons to other qualifying VAMCs (Supplemental Figure 1). Each Chief was asked to forward this e-mail to colleagues. In all, six feedback e-mails were sent to each site.
- The Chief of Urology was also asked to serve as a local champion, *ie*, to encourage peers to better adhere to AUA guidelines on antimicrobial prophylaxis.^16^ The champion was not trained or compensated for this role.

The graphs included in each feedback e-mail were iteratively designed prior to the trial’s initiation by the main study team, which included two urologists (R.L.S. and V.T.P.). Once the baseline dataset was created and validated, updating the dataset and preparing the graphs took approximately 8 hours of the data analyst’s (Q.S.) time once every 2 months. Data were collected both retrospectively and prospectively from the VHA Corporate Data Warehouse (CDW) via the VHA Informatics and Computing Infrastructure.

Because the feedback reports were demonstrating no change in post-procedural antimicrobial use at two participating sites, a follow-up virtual meeting was held with each Chief of Urology during January 2023 to ensure there was fidelity to the intervention and to assess for barriers to improvement. In response to this meeting, no additional actions were taken at site 1. At site 2, the Chief of Urology acknowledged not routinely sending feedback to the staff and agreed to start doing so. At site 3, an e-mail reminder to all urology residents encouraging adherence to AUA guidelines was sent.

### Outcomes

The primary outcome was excess post-procedural antimicrobial use, defined as the prescription of an antimicrobial agent that the patient received on the date after the surgical procedure (Supplemental Table 1).^3,4^ For the primary outcome, we only captured antimicrobials recommended by the AUA for peri-procedural prophylaxis as well as other agents with good coverage of uropathogens (Supplemental Table 2). Patients were eligible for this outcome if they underwent a qualifying procedure (ureteroscopy, TURP, TURBT), which were identified based on our prior methods (Supplemental Table 3).^4^ In patients who were exposed to excess post-procedural antimicrobials, the duration of post-procedural antimicrobial use was captured by counting the number of continuous days of inpatient, bar-coded antimicrobial administration, if relevant, and by using the days-supply field for outpatient prescriptions.

Secondary outcomes were measured to assess the safety of the intervention. Unplanned return visits were defined as an Emergency Department visit or hospital readmission to an acute-care bed at a VHA facility for any indication within 30 days of the patient’s index urologic procedure, excluding the admission associated with the index procedure. Late antimicrobial prescriptions were defined as the prescription of a designated antimicrobial by any provider between 7–30 days after the date of the patient’s urologic procedure. *Clostridioides difficile* testing within 30 days of the patient’s urologic procedure and all-cause death within 30 days of the patient’s index urologic procedure were also assessed.

### Data analysis

Observations occurring within the implementation phase (July 2022) were omitted from all analyses. To ensure that the effect of the intervention at each site was separately evaluated, observations from each of the three sites were stratified into separate data sets.

To model the primary outcome, we used a logistic regression model and a set of three explanatory variables: time, a binary indicator for an observation occurring within the intervention phase, and an interaction term between these two main effects (time and intervention). Time was measured in days divided by 365, giving an annualized figure for the time coefficient estimate and interaction coefficient. The median duration of post-procedural antimicrobial prescriptions at each site were compared using the Wilcoxon rank-sum test. To model the secondary outcomes of unplanned return visits and late antimicrobial prescriptions, we used a similar approach to what is described above with the addition of a fourth explanatory variable (a binary indicator for the primary outcome) to the logistic regression model. This last variable was included to assess the risk of these secondary outcomes in patients who were not exposed to post-procedural antimicrobials compared to those who were exposed.

### Protection of human subjects

The Institutional Review Board (IRB) at the University of Iowa and as well as the Research and Development Committee of the Iowa City approved this study and waived written informed consent. All other sites were deemed to not be engaged in research activities, so seeking local IRB approval was left to the local team’s discretion.

## Results

During the baseline period, there were 1,272 qualifying procedures performed across the intervention sites compared to 525 during the intervention period; the total number of procedures across both periods was therefore 1,797. In total, 535 (29.8%) procedures were performed while inpatient with a median hospital stay of 2 days (interquartile range, IQR 2–3). Characteristics of patients at each site are shown in Table 1. The median patient-age across all 1,797 procedures was 73 years (IQR 67–77), and 1,738 (96.7%) were males. The most common procedure across both periods was TURBT (56.4%) followed by ureteroscopy (21.9%) and TURP (21.7%).

**Table 1.** Characteristics of patients across 3 intervention sites during both the baseline and intervention periods

	Total n = 1797	Site 1		Site 2		Site 3	
		Baseline n = 841	Intervention n = 338	Baseline n = 110	Intervention n = 76	Baseline n = 321	Intervention n = 111
**Age, median (IQR)**	73 (67–77)	73 (67–77)	73 (66–77)	74 (70–81)	75 (69–81)	72 (67–77)	75 (70–79)
**Male gender**	1738 (96.7)	806 (95.8)	327 (96.8)	107 (97.3)	74 (97.4)	315 (98.1)	109 (98.2)
**Race**							
White	1404 (78.1)	657 (78.1)	245 (72.5)	66 (60.0)	29 (38.2)	303 (94.4)	104 (94.7)
Black	252 (14.0)	121 (14.4)	58 (17.2)	29 (26.4)	35 (46.1)	5 (1.6)	4 (3.6)
Other	141 (7.9)	63 (7.5)	35 (10.4)	15 (13.6)	12 (15.8)	13 (4.1)	3 (2.7)
**Hispanic ethnicity**	55 (3.1)	11 (1.3)	5 (1.5)	21 (19.1)	13 (17.1)	4 (1.3)	1 (0.9)
**Comorbidities**							
CHF	234 (13.0)	99 (11.8)	49 (14.5)	12 (10.9)	10 (13.2)	50 (15.6)	14 (12.6)
COPD	535 (29.8)	248 (29.5)	108 (32.0)	34 (30.9)	24 (31.6)	86 (26.8)	35 (31.5)
Diabetes mellitus	755 (42.0)	376 (44.7)	134 (39.6)	35 (31.8)	40 (52.6)	120 (37.4)	50 (45.1)
Metastatic cancer	74 (4.1)	41 (4.9)	13 (3.9)	4 (3.6)	6 (7.9)	5 (1.6)	5 (4.5)
Neurologic disorder	90 (5.0)	35 (4.2)	19 (5.6)	5 (4.6)	2 (2.6)	19 (5.9)	10 (9.0)
Obesity	474 (26.4)	233 (27.7)	99 (29.3)	25 (22.7)	24 (31.6)	77 (24.0)	16 (14.4)
Renal disease	280 (15.6)	120 (14.3)	44 (13.0)	16 (14.6)	22 (29.0)	67 (20.9)	16 (14.4)
**Procedure**							
TURBT	1013 (56.4)	536 (63.7)	177 (52.4)	48 (43.6)	43 (56.6)	147 (45.8)	62 (55.9)
TURP	390 (21.7)	142 (16.9)	78 (23.1)	33 (30.0)	26 (34.2)	85 (26.5)	26 (23.4)
Ureteroscopy	394 (21.9)	163 (19.4)	83 (24.6)	29 (26.4)	7 (9.2)	89 (27.7)	23 (20.7)
**Hospitalized for procedure**	535 (29.8)	255 (30.3)	134 (39.6)	44 (40.0)	30 (39.5)	57 (17.8)	15 (13.5)

Abbreviation: COPD, chronic obstructive pulmonary disease; CHF, congestive heart failure; TURBT, transurethral resection of a bladder tumor; TURP, transurethral resection of the prostate

### Changes in post-procedural antimicrobial use

Across all 3 sites, 644 (50.6%) patients received excess post-procedural antimicrobials during the baseline period compared to 216 (41.1%) during the intervention period. For the baseline period, 219 (34.0%) of the patients received excess antimicrobials while inpatient compared to 93 (43.1%) during the intervention period. Supplemental Table 4 shows the frequency of excess post-procedural antimicrobial use based on site and procedure.

**Figure 1.**
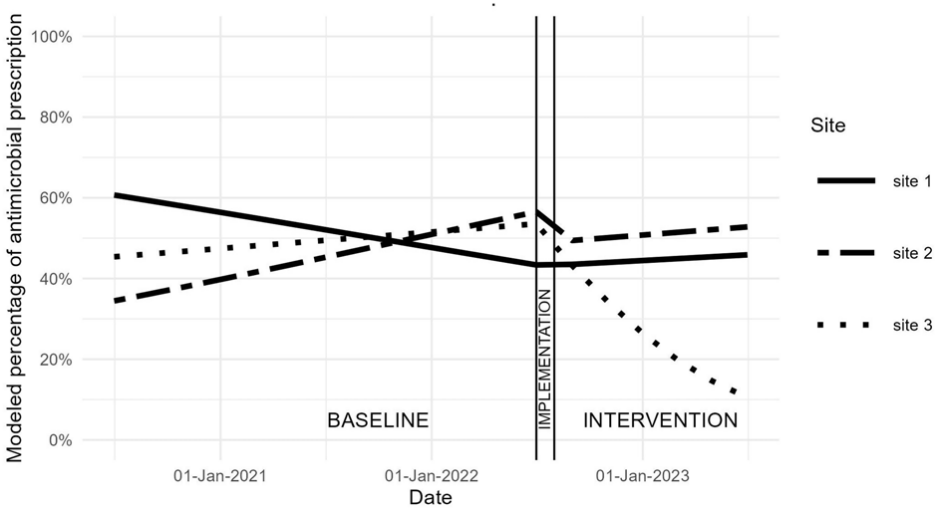
Modeled percentage of excess post-procedural antimicrobial use after common endourologic procedures across 3 participating hospitals during the baseline and intervention periods.

The modeled percentage of post-procedural antimicrobial use over time is shown in Figure 1, and the findings of the statistical analysis are shown in Table 2. At site 1, there was a decreasing odds of post-procedural antimicrobial use over the baseline period (OR 0.70, 95% CI 0.55–0.90) that did not significantly change after the intervention started. At site 2, there was no change in excess post-procedural antimicrobial use during the baseline period or after the intervention began. At site 3, there was no change in excess post-procedural antimicrobials during the baseline period or immediately after the intervention started, but with increasing time, the odds of prescribing a post-procedural antimicrobial significantly decreased relative to the baseline time trend (OR 0.09; 95% CI 0.02–0.45). This OR equates to a 91% annualized decrease in the odds of a patient receiving a post-procedural antimicrobial prescription during the intervention period relative to the baseline time trend (*P* < 0.01).

**Table 2.** Logistic regression results for the primary outcome (excess post-procedural antimicrobial use) adjusting for the effect of the intervention and procedure date while stratifying by site

	Odds ratio (multiplicative)	95% confidence interval for effect on odds	*P*-value of effect
**Site 1**			
Baseline time effect (years)	0.70	0.55–0.90	<0.01
Intervention immediate effect	0.99	0.56–1.74	0.96
Intervention phase time effect	1.59	0.71–3.59	0.26
**Site 2**			
Baseline time effect (years)	1.57	0.78–3.26	0.21
Intervention immediate effect	0.73	0.18-2.89	0.65
Intervention phase time effect	0.75	0.14–4.15	0.74
**Site 3**			
Baseline time effect (years)	1.21	0.81–1.72	0.39
Intervention immediate effect	0.97	0.33–2.81	0.95
Intervention time effect	0.09	0.02–0.45	<0.01

For patients who did receive post-procedural antimicrobials, there was a statistically significant increase in post-procedural antimicrobial duration during the intervention period compared to the baseline period at site 1 [median 3 (IQR 3–5) versus 4 (IQR 3–6), *P* = 0.01] and site 3 [median 3 (IQR 2–3) versus 3 (IQR 3–6.5); *P* < 0.01] but not at site 2 [median 3 (IQR 1–5) versus 4 (IQR 3–5); *P* = 0.14]

### Secondary outcomes

The unadjusted frequency of unplanned visits across all sites changed from 14.6% to 21.5% between the baseline and intervention periods while late antimicrobial prescriptions changed from 13.3% to 15.8%. These outcomes differed when a post-procedural antimicrobial was prescribed (Table 3) and by site (Supplemental Table 5). In the adjusted analysis, there was no significant change in either safety outcome during the baseline period or after the intervention began at any site (Tables 4 and 5). As shown in Supplemental Table 6, the adjusted odds of an unplanned visit across all sites was lower when a post-procedural antimicrobial was not prescribed compared to when it was prescribed (OR 0.68, 95% CI 0.53–0.87). Across all three sites, the adjusted odds of a late antimicrobial prescription was also significantly lower when a post-procedural antimicrobial was not prescribed compared to when it was prescribed (OR 0.76, 95% CI 0.58–0.99).

**Table 3.** Unadjusted data on unplanned visits and late antimicrobial prescriptions during the baseline and intervention periods, stratified by whether or not a post-procedural antimicrobial was prescribed

	Post-procedural antimicrobial given		No post-procedural antimicrobial given	
	Baseline period n = 644	Intervention period n = 216	Baseline period n = 628	Intervention period n = 309
Unplanned visits				
ED visit	96 (14.9)	52 (24.1)	71 (11.3)	45 (14.6)
Hospitalization	46 (7.1)	26 (12.0)	37 (5.9)	25 (8.1)
ED and/or hospitalization	106 (16.5)	58 (26.9)	80 (12.7)	55 (17.8)
Late antimicrobial prescription	97 (15.1)	37 (17.1)	72 (11.5)	46 (14.9)

Abbreviation: ED, Emergency Department

**Table 4.** Logistic regression results stratified by site for unplanned visits after adjusting for time, the intervention, and whether or not a post-procedural antimicrobial was prescribed

	OR Estimate	95% CI for OR	*P*-value
**Site 1**			
Baseline time effect (years)	1.20	0.84–1.72	0.32
Baseline post-procedural antimicrobial effect*	0.60	0.39–0.90	0.01
Intervention immediate effect	1.35	0.59–3.04	0.46
Intervention phase time effect (years)	0.84	0.30–2.40	0.74
Post-procedural antimicrobial effect during intervention*	0.94	0.47–1.88	0.87
**Site 2**			
Baseline time effect (years)	1.03	0.44–2.39	0.94
Baseline post-procedural antimicrobial effect*	0.56	0.23–1.34	0.19
Intervention immediate effect	0.59	0.11–3.02	0.53
Intervention phase time effect (years)	4.16	0.66–28.52	0.14
Post-procedural antimicrobial effect during intervention*	1.41	0.38–5.16	0.61
**Site 3**			
Baseline time effect (years)	1.11	0.65–1.89	0.70
Baseline post-procedural antimicrobial effect*	1.25	0.67–2.34	0.49
Intervention immediate effect	2.36	0.56–9.30	0.23
Intervention phase time effect (years)	0.48	0.08–2.95	0.43
Post-procedural antimicrobial effect during intervention*	0.59	0.17–2.23	0.42

*The adjusted odds of the relevant outcome when a post-procedural antimicrobial was not prescribed compared to the odds of the relevant outcome with a post-procedural antimicrobial was prescribed.

**Table 5.** Logistic regression results stratified by site for late antimicrobial prescriptions after adjusting for time, the intervention, and whether or not a post-procedural antimicrobial was prescribed

	OR Estimate	95% CI for OR	*P*-value
**Site 1**			
Baseline time effect (years)	1.15	0.80–1.66	0.46
Baseline post-procedural antimicrobial effect*	0.62	0.40–0.94	0.03
Intervention immediate effect	1.01	0.42–2.38	0.98
Intervention phase time effect (years)	0.97	0.33–2.93	0.96
Post-procedural antimicrobial effect during intervention*	1.36	0.67–2.79	0.39
**Site 2**			
Baseline time effect (years)	1.81	0.76–4.43	0.18
Baseline post-procedural antimicrobial effect*	1.13	0.44–2.95	0.80
Intervention immediate effect	0.35	0.15–1.28	0.27
Intervention phase time effect (years)	1.15	0.14–10.24	0.89
Post-procedural antimicrobial effect during intervention*	0.95	0.22–4.08	0.95
**Site 3**			
Baseline time effect (years)	1.33	0.76–2.35	0.32
Baseline post-procedural antimicrobial effect*	0.83	0.43–1.60	0.58
Intervention immediate effect	0.22	0.02–1.41	0.15
Intervention phase time effect (years)	2.27	0.22–30.19	0.51
Post-procedural antimicrobial effect during intervention*	1.40	0.27–10.94	0.71

*The adjusted odds of the relevant outcome when a post-procedural antimicrobial was not prescribed compared to the odds of the relevant outcome with a post-procedural antimicrobial was prescribed.

Testing for *C. difficile* during the 30-day post-procedural period was rare during both periods (0.5% vs. 0.2%). Mortality during the 30-days after the procedure was also rare during both the baseline (0.3%) and intervention periods (0.8%).

## Discussion

Implementation of our bundled intervention was associated with reduced post-procedural antimicrobial use after urologic procedures at only one of the three participating sites. The site with reduced post-procedural antimicrobial use did not have more frequent unplanned visits or other antimicrobial use during the 30 days after the index procedure. These mixed findings support the safety but also highlight the difficulty of implementing AUA guidelines on surgical antimicrobial prophylaxis.

Given the frequency of post-procedural antimicrobial use, the lack of benefit, and the potential for antimicrobial-related harm, there is a need to develop strategies to minimize this practice. Our study suggests that a bundled approach using audit-and-feedback with peer-to-peer comparisons, education and a local champion may be an effective means for changing antimicrobial-prescribing behavior in some settings. Based on the design of our study, it is not possible to know which of these strategies was most influential at site 3. We speculate that resident-physician involvement in the upfront meeting, a unique feature of site 3, may have more effectively disseminated knowledge and helped the local champion reset local norms. Though the bundle was effective at site 3, the difficulty we had in recruiting sites (*eg*, only 3 of 13 agreed to participate) could be a barrier to more widespread adoption of these strategies.

We suspect that there were several reasons why this bundled approach was not effective at sites 1 and 2. First, there may have been skepticism regarding the quality and strength of the data behind the AUA guidelines or the applicability of these guidelines to the patient populations treated at these sites. At certain sites, there may have been the perception that the local patient population was too complex for the guidelines to apply. In addition, site 2’s lack of feedback dissemination during the first 6 months of the project likely also played a role in that site’s lack of change. At both sites 1 and 2, urology residents did not attend our initial virtual meeting, and site 2 had the lowest number of any participants at this session. It may have been especially important to incorporate residents into the initial education, as residents frequently are the prescribers of peri-procedural antibiotics in these types of endourologic cases.

With any new healthcare innovation, potential unintended consequences should be considered. Our trial did not find evidence of significantly worse clinical outcomes during the intervention period at any of the participating sites, including at site 3. We cannot be certain why there was an apparent discrepancy between the unadjusted and adjusted analyses for unplanned visits and late antimicrobial prescriptions, but changes in care delivery due to the COVID-19 pandemic may have played a role. Notably, patients who were not prescribed a post-procedural antimicrobial were significantly less likely to have either of these outcomes. While the percentage of patients exposed to post-procedural antimicrobials decreased at site 3, the median duration of post-procedural antimicrobials increased. This may reflect withholding short courses of unnecessary post-procedural antimicrobials in patients who would not benefit from them and prescribing full therapeutic course in situations when they were indicated (*eg*, infection).

An unexpected finding in our study is that patients not prescribed post-procedural antimicrobials were less likely to have unplanned visits or to receive late antimicrobial prescriptions. While the reasons for this association are unclear, a few explanations seem plausible. One potential explanation is that some urologists were prescribing post-procedural antimicrobials to patients with more comorbidities who, in turn, were at higher risk of any complications, including the need for unplanned post-procedural care. In addition, it is possible that some post-procedural antimicrobials were causing adverse events that led to unplanned visits, thereby increasing the relative frequency of these visits compared to patients who did not receive post-procedural antimicrobials. To understand which of these mechanisms are contributing, further research is warranted.

This study has some limitations that should be acknowledged. First, the study was not randomized, and we cannot exclude the possibility that temporal confounding or regression to the mean explained the positive changes seen at site 3. Second, the lack of fidelity to the intervention at site 2 makes it difficult to assess the effectiveness of the intervention in that setting. Third, while our primary outcome does not distinguish necessary versus unnecessary post-procedural antimicrobial use, a prior study found that 85% of post-procedural antimicrobials were not indicated, based on expert review of 375 patients’ medical records.^3^ Fourth, our secondary outcomes do not reflect any care delivered outside the VA system, so certain adverse events could have been missed. However, there is not a good reason to suspect that the use of VA versus non-VA care during the post-procedural period would have substantially differed between the baseline and intervention periods. Fifth, the intervention only lasted 12 months, so the sustainability of delivering feedback over the long-term is unclear. Site 3 may have regressed to its baseline once the intervention stopped; this has been observed with other audit-and-feedbacks interventions that aimed to de-implement unnecessary antimicrobials.^17,18^ Sixth, our analysis of the secondary outcomes did not adjust for the presence of comorbidities, which may have influenced the decision to prescribe post-procedural antimicrobials and may have also been associated with the safety outcomes. The important finding relative to this trial is that these secondary outcomes did not increase at site 3 as post-procedural antimicrobials decreased. Seventh, we were unable to measure how many urologists saw the feedback reports and whether these reports were acceptable. We did measure other implementation outcomes, including adoption and fidelity.^19^ Finally, this study was performed within the VA system and may not be generalizable to non-VA settings.

In conclusion, we have shown that a bundled intervention was associated with reductions in post-procedural antimicrobial use for urologic procedures at 1 of the 3 participating sites. These results support the safety of stewardship but also highlight the difficulty of implementing prescribing patterns more in line with AUA guidelines. Future studies should consider optimizing the implementation of this bundle and/or incorporating additional strategies to better address local barriers to improvement.

## Supporting information

Livorsi et al. supplementary material Livorsi et al. supplementary material

## Data Availability

The data sets generated during and/or analyzed during the current study are not publicly available due to Department of Veterans Affairs rules.

## Supplementary material

The supplementary material for this article can be found at https://doi.org/10.1017/ice.2024.172
