# Supplementary material for: A pilot intervention trial to reduce the use of post-procedural antimicrobials after common endourologic surgeries
Source: Infect Control Hosp Epidemiol. 2024 Nov 7;46(1):50–6. doi: 10.1017/ice.2024.172 (PMC11717473; doi:10.1017/ice.2024.172)
Supplement: Livorsi et al. supplementary material [file S0899823X24001727sup001.docx]

**Supplemental Material: Table of Contents**

| **Table or figure** | **Page number** |
| --- | --- |
| Supplemental Figure 1. Feedback report on post-procedural antimicrobial use for common urologic procedures | 2 |
| Supplemental Table 1. Definition of excess post-procedural antibiotic use | 3 |
| Supplemental Table 2. Recommended Antimicrobial Prophylaxis for Urologic Procedures According to American Urological Association Guidelines 2019 | 4 |
| Supplemental Table 3. CPT and ICD-10 codes used to identify qualifying urologic procedures | 5 |
| Supplemental Table 4. Excess post-procedural antimicrobial use based on type of procedure during the baseline and intervention periods, stratified by site | 6 |
| Supplemental Table 5. Unplanned visits and late antimicrobial prescriptions during the baseline and intervention periods, stratified by site | 7 |
| Supplemental Table 6. Odds ratio of unplanned visits and late antimicrobial prescriptions after adjusting for procedure date, baseline vs. intervention period, and whether a post-procedural antimicrobial was prescribed | 8 |

**Supplemental Figure 1. Feedback report on excess post-procedural antimicrobial use for common urologic procedures**

**Supplemental Table 1.** **Definition of excess post-procedural antibiotic use**^1,2^

| **Criterion** | **Location of antibiotic prescription** | **Timing of antibiotic prescription** | **Days-supply of the antibiotic prescribed** |
| --- | --- | --- | --- |
| 1 | Inpatient | Day after the procedure | ≥ 1 |
| 2 | Outpatient | Day after the procedure | ≥ 1 |
| 3 | Outpatient | Day prior to the procedure^3^ | ≥ 2 |
| 4 | Outpatient | Day of the procedure | ≥ 2 |
| 5 | Outpatient | 2-30 days prior to the procedure | Duration extended beyond the procedure date |

1. If criterion 1 was not met, we evaluated criterion 2. If either criterion 1 nor 2 were not met, we evaluated criterion 3. If criterion 1-3 were not met, we evaluated criterion 4, and so on.

2. Antibiotics that qualified for this definition are shown in Supplemental Table 2.

3. We assumed the patient was instructed to start the antibiotic on the procedure date.

**Supplemental Table 2.** **Recommended Antimicrobial Prophylaxis for Urologic Procedures According to American Urological Association Guidelines 2019**

| **Procedure** | **Prophylaxis Indicated** | **Antimicrobial of choice**^1^ | **Alternative antimicrobials^1^** | **Duration of therapy** |
| --- | --- | --- | --- | --- |
| **TURBT, TURP, URS** | All patients | Fluoroquinolone, sulfamethoxazole-trimethoprim | Aminoglycosides +/- ampicillin, 1^st^/2^nd^ generation cephalosporin, amoxicillin/clavulanate, aztreonam +/- ampicillin | Single pre-procedural dose^2^ |

Abbreviations: TURBT = transurethral resection of the bladder, TURP = transurethral resection of the prostate, URS = ureteroscopy.

1. The study’s primary outcome of excess post-procedural antimicrobial use captured the antimicrobials listed in this table as well as other antimicrobials with coverage of uro-pathogens. These additional agents included: amoxicillin, ampicillin-sulbactam, carbapenem, 3^rd^/4^th^ cephalosporins, clindamycin, daptomycin, fluconazole, fosfomycin, piperacillin-tazobactam, linezolid, nitrofurantoin, and vancomycin.

**Supplemental Table 3. CPT and ICD-10 Codes used to identify qualifying urologic procedures**

| **Procedure** | **CPT codes** | **ICD-10 codes** |
| --- | --- | --- |
| **TURP** | 52601, 52450, 52630 | 0VB03ZX, 0VB03ZZ, 0VB04ZX, 0VB04ZZ, 0VB07ZX, 0VB07ZZ, 0VB08ZX, 0VB08ZZ, 0VT04ZZ, 0VT07ZZ, 0VT08ZZ, 0V503ZZ, 0V504ZZ, 0V507ZZ, 0V508ZZ |
| **TURBT** | 52224, 52234, 52235, 52240 | 0T5B7ZZ, 0T5B8ZZ, 0T5C7ZZ, 0T5C8ZZ, 0TBB7ZX, 0TBB7ZZ, 0TBB8ZX, 0TBB8ZZ, 0TBC7ZX, 0TBC7ZZ, 0TBC8ZX, 0TBC8ZZ |
| **URS^1^** | 52343, 52344, 52345, 52346, 52351, 52352, 52353, 52354, 52355 | 0TC37ZZ, 0TC38ZZ, 0TC47ZZ, 0TC48ZZ, 0TC67ZZ, 0TC68ZZ, 0TC77ZZ, 0TC78ZZ, OTJ93ZZ, 0TJ97ZZ, 0TJ94ZZ, 0TJ98ZZ |

Abbreviations: CPT = Current Procedural Terminology; ICD-10 = International Classification of Diseases, 10^th^ revision: TURBT = transurethral resection of bladder tumor, TURP = transurethral resection of the prostate, and URS = ureteroscopy.

^1^Ureteroscopies with or without urinary stones were included.

**Supplemental Table 4. Excess post-procedural antimicrobial use based on type of procedure during the baseline and intervention periods, stratified by site**

|  | **Baseline**  Number who received excess post-procedural antimicrobials / total procedures (%) | **Intervention**  Number who received excess post-procedural antimicrobials / total procedures (%) |
| --- | --- | --- |
| **Site 1**  TURBT  TURP  Ureteroscopy  Total | 222/536 (**41**)  107/142 (**75**)  108/163 (**66**)  437/841 (**52**) | 47/177 **(27**)  46/78 (**59**)  58/83 (**70**)  151/338 (**45**) |
| **Site 2**  TURBT  TURP  Ureteroscopy  Total | 19/48 (**40**)  15/33 (**46**)  15/29 (**52**)  49/110 (**45**) | 22/43 (**51**)  14/26 (**54**)  3/7 (**43**)  39/76 (**51**) |
| **Site 3**  TURBT  TURP  Ureteroscopy  Total | 56/147 (**38**)  61/85 (**72**)  41/89 (**46**)  158/321 (**49**) | 15/62 (**24**)  7/26 (**27**)  4/23 (**17**)  26/111 (**23**) |

Abbreviation: TURBT transurethral resection of a bladder tumor; TURP transurethral resection of the prostate

**Supplemental Table 5. Unplanned visits and late antimicrobial prescriptions during the baseline and intervention periods, stratified by site**

|  | **Site 1** | | **Site 2** | | **Site 3** | | **All** | |
| --- | --- | --- | --- | --- | --- | --- | --- | --- |
|  | **Baseline**  **n=841** | **Intervention**  **n=338** | **Baseline**  **n=110** | **Intervention**  **n=76** | **Baseline**  **n=321** | **Intervention**  **n=111** | **Baseline**  **n=1272** | **Intervention**  **n=525** |
| **Unplanned visits**  ED  Hospitalization  Either ED and/or hospital | 101 (12.0)  52 (6.2)  112 (13.3) | 58 (17.2)  28 (8.3)  64 (18.9) | 27 (24.6)  9 (8.2)  27 (24.6) | 27 (35.5)  13 (17.1)  29 (38.2) | 39 (12.2)  22 (6.9)  47 (14.6) | 12 (10.8)  10 (9.0)  20 (18.0) | 167 (13.1)  83 (6.5)  186 (14.6) | 97 (18.5)  51 (9.7)  113 (21.5) |
| **Late antimicrobial prescription** | 105 (12.5) | 56 (16.6) | 23 (20.9) | 16 (21.1) | 41 (12.8) | 11 (9.9) | 169 (13.3) | 83 (15.8) |

Abbreviation: ED Emergency Department

**Supplemental Table 6. Odds ratio of unplanned visits and late antimicrobial prescriptions after adjusting for procedure date, baseline vs. intervention period, and whether a post-procedural antimicrobial was prescribed**

| **Secondary outcome** | **Odds ratio*** | **95% confidence interval**  **for Odds Ratio** | **p-value** |
| --- | --- | --- | --- |
| **Unplanned visit** |  |  |  |
| Site 1 | 0.58 | 0.42-0.81 | <0.01 |
| Site 2 | 0.65 | 0.34-1.24 | 0.19 |
| Site 3 | 1.11 | 0.64-1.92 | 0.72 |
| All sites | 0.68 | 0.53-0.87 | <0.01 |
| **Late antimicrobial prescription** |  |  |  |
| Site 1 | 0.69 | 0.49-0.97 | 0.03 |
| Site 2 | 1.11 | 0.54-2.27 | 0.78 |
| Site 3 | 0.87 | 0.48-1.60 | 0.65 |
| All sites | 0.76 | 0.58-0.99 | 0.04 |

*The adjusted odds of the relevant outcome when a post-procedural antimicrobial was not prescribed compared to the odds of the relevant outcome with a post-procedural antimicrobial was prescribed.
